# Determinants of success in treating acute ischemic cerebral and ocular ischemia through carotid revascularization. An observational study of a case series

**DOI:** 10.1590/0100-6991e-20223400-en

**Published:** 2022-11-10

**Authors:** ARNO VON RISTOW, MARCOS SANTOS, ALBERTO VESCOVI, BERNARDO MASSIÈRE, BRUNO DEMIER, PEDRO SARTORI, PAULO NIEMEYER

**Affiliations:** 1 - Pontifícia Universidade Católica do Rio de Janeiro, Pós-graduação de cirurgia vascular e endovascular - Rio de Janeiro - RJ - Brasil; 2 - Universidade Federal do Rio de Janeiro, Instituto de Ciências Biomédicas, Laboratório de Morfogênese Celular - Rio de Janeiro - RJ - Brasil; 3 - Instituto Estadual do Cérebro Paulo Niemeyer - Rio de Janeiro - RJ - Brasil

**Keywords:** Carotid Stenosis, Ischemic Stroke, Endarterectomy, Carotid, Carotid Artery Diseases, Ischemic Attack, Transient, Estenose das Carótidas, Acidente Vascular Cerebral, Endarterectomia das Carótidas, Doenças das Artérias Carótidas, Ataque Isquêmico Transitório

## Abstract

**Objective:**

stroke etiology is ischemia in 85%, and in circa 25% of these, the source is the extracranial carotid. Recurrence is frequent and usually more severe. Carotid revascularization prevents new ischemic strokes. The sooner the treatment is undertaken, complete recovery chances are greater with less recurrences. But, historically, intervention in the acute setting was catastrophic. Objective: Identify determinants of success when carotid revascularization after a recent cerebral ischemic event is performed.

**Materials and Methods:**

A cohort of 50 subjects underwent carotid revascularization after ischemic symptoms, within a period of 71 months. The currently diagnostic tools were used, and the symptoms stratified by the Rankin scale. The extension of the cerebral lesion and the source location the source of the event was analyzed.

**Results:**

indications were based on the Rankin Scale (R0: 35.4%; R1: 45.8%; R2:18.8% and R3: zero), on the location of the source and the absence of ischemic areas greater than 15mm. An early surgical approach was adopted in all patients. Extreme care was applied to control arterial pressure. At discharge, no additional deficits were observed.

**Conclusions:**

carotid revascularization after ischemic events can be achieved without additional morbidity and no recurrences, using the most appropriate therapy in the shortest time, in patients with Rankin Scale up to 2, absence of intracranial hemorrhage and single or multiple ischemic intracerebral areas, with 15mm or less in their greater dimension.

## RESUMO

**Introdução:**

isquemia é a etiologia do acidente vascular cerebral em 85% dos casos e em cerca de 25% destes, a fonte é a carótida extracraniana. Recorrência é frequente e usualmente mais grave que a inicial. A revascularização carotídea previne novos acidentes. Quanto mais cedo for realizado o tratamento, maiores as chances de recuperação e menor o risco de recorrência. Mas, historicamente, os resultados das intervenções precoces eram catastróficos.

**Objetivos:**

identificar determinantes de sucesso da revascularização carotídea após um evento isquêmico cerebral recente.

**Materiais e Métodos:**

uma coorte de 50 pacientes foi submetida à revascularização carotídea após sintomas isquêmicos, em um período de 71 meses. Foram empregados os métodos de investigação atuais e os sintomas estratificados pela Escala de Rankin. A extensão das lesões cerebrais e a fonte do evento foram estudados e analisados.

**Resultados:**

as indicações foram baseadas na escala de Rankin (R0: 35.4%; R1: 45.8%; R2: 18.8% e R3: zero), na localização da fonte e na ausência de áreas isquêmicas com menos de 15mm. Uma abordagem cirúrgica precoce foi empregada em todos os pacientes. Cuidados extremos com a pressão arterial foram aplicados. Na alta hospitalar, nenhum déficit adicional foi observado.

**Conclusões:**

a revascularização carotídea após eventos isquêmicos pode ser realizada sem morbidade adicional ou recorrências, empregando a terapêutica mais apropriada no período de tempo mais curto, em pacientes classificados como Rankin até 2, na ausência de hemorragia intracraniana e com áreas isquêmicas intracerebrais únicas ou múltiplas, com menos de 15mm em sua maior dimensão.

## INTRODUCTION

Stroke is among the leading causes of hospitalizations, disabilities, and death. In fact, stroke is the second leading cause of death globally^1^ ^,^ ^2^.

In 1970, the World Health Organization (WHO) defined stroke as “rapidly developing clinical signs of focal (or global) disturbance of cerebral function lasting more than 24 hours with no apparent cause other than of vascular origin”^3^. However, over the last 51 years, several advances have been developed in clinical assessment, imaging exams, and treatment of stroke. Therefore, other classifications have been proposed^4^.

Stroke is a disease characterized by a sudden development of a neurological deficit^5^. It arises from changes in the arterial supply of the brain, which in turn, causes blockages of both nutrients and oxygen supply to the nervous tissue. Based on its etiology, it may be further classified into two general categories, namely ischemic or hemorrhagic stroke. However, the clinical presentation of both ischemic and hemorrhagic strokes is often very similar. Noteworthy, on many occasions such similarities are clinically relevant, since they can be confounding factors during in the diagnosis process^6^.

In 1993, the ORG 10172 Trial on Acute Stroke Treatment (TOAST) introduced a classification for ischemic strokes, based on its etiology. According to this classification, five subtypes ischemic strokes can be defined. Those are: large-artery-atherosclerosis (embolus/thrombosis); cardioembolism; small-vessel occlusion (lacunes); stroke of other determined etiology and stroke of undetermined etiology^7^. 

Notwithstanding many advances in the diagnosis and treatment of both ischemic and hemorrhagic stroke have been witnessed in the last decades, there are still several questions that must be addressed. For instance, regarding the available surgical approaches to treat ischemic stroke, the benefits of carotid endarterectomy (CE) in preventing the occurrence of cerebrovascular events are unquestionable^5^ ^,^ ^8^. However, the most appropriate moment to use this approach after an ischemic stroke is still in debate. In fact, the main factors that affect the success of the surgical approaches after the occurrence of an ischemic stroke is yet to be clearly defined. 

The aim of this study is to determine the effectivity, safety and death rates of carotid revascularization used to treat acute cerebrovascular ischemic events, including transient ischemic attacks (TIA) and ischemic strokes (IS) of confirmed carotid etiology, early after the index event, in patients with no detectable cerebral infarction or with cerebral infarction areas up to 15mm in its major diameter. We postulate that the extension of the cerebral ischemic lesion is a vital determinant to decide the ideal time for the carotid revascularization.

## METHODS

This observational-exploratory study evaluated 50 subjects that underwent carotid revascularization after ischemic stroke, evaluated and treated in the following tertiary hospitals: Copa Star, Copa D’Or and Quinta D’Or, in Rio de Janeiro, Brazil, within a period of 71 months (from August 2014 to June 2020). Supervision of this study was supplied by the Instituto Estadual do Cérebro Paulo Niemeyer, from the Secretaria de Estado de Saúde, Rio de Janeiro, Brazil. This study had the approval nr. 4.701.956 of the Institutional Ethics Committee. All data was collected prospectively and stored in our archives, for later study and analysis. All patients were stratified by the modified Rankin score (mRs) at admittance and discharge; patients with mRs grade 4 and above were excluded of this study^9^. All patients were treated according to the Albany Protocol: stratification of the neurological deficit according to the mRs (selecting patients with symptoms ranging from 0 to 3); to exclude intracranial hemorrhage and evaluate ischemic changes, computerized tomography (CT) and/or diffusion weighted magnetic resonance imaging (DWI) was obtained. In cases with suspect carotid disease, immediate computerized angiography (CTA), carotid duplex scanning and echocardiography were performed. Selected patients underwent thrombolysis and vascular surgery opinion was asked in cases of carotid stenosis above 50%^10^ ^,^ ^11^. An individualized analysis of each patient, using all available medical resources was applied. The extension of the cerebral parenchymal lesion, which was individually evaluated, as well as the precise location of the source of the event and the absence of single or multiple ischemic areas greater than 15mm in its greatest extent, evidenced by CT or DWI. The diagnosis, clinical conduct and all surgical procedures were performed directly by the main author of the study or supervised, coordinated, and assisted by him. The carotids were revascularized either by direct surgical approach by longitudinal or eversion endarterectomies, segmental resection and end-to-end anastomosis or by endovascular means^12^ ^,^ ^15^. 

Demographics, known risk factors for cerebrovascular disease, focal and non-focal symptoms, characteristics of the carotid disease, presence and size of cerebral ischemic changes determined by CT and/or DWI; the degree of carotid stenosis as well as the constitution of the atheromatous plaques were analyzed, as well as the interval between the index event and the effective revascularization and the type of revascularization was evaluated. Patients with ischemic foci of more than 15mm in their largest length were excluded from the study. Finally, 30-days general and neurological complications were evaluated. Brain parenchyma imaging exams obtained during the ischemic event, prospectively stored, were reevaluated in July 2020, to check the presence and extent of ischemic areas. Of the 50 patients of the study, 47 underwent cerebral parenchymal imaging studies at admission. The three patients that were not evaluated by CT nor MRI had presented isolated transient vision loss related to the ischemia (amaurosis fugax).

The descriptive statistical analysis was carried out using the software Stata 16.1 (StataCorp, College Station, TX, USA). Institution Ethics Committee Approval Nr: 4.701.956, May 10^th^, 2021.

## RESULTS

The pre-surgical and post-surgical data related to the 50 patients that underwent revascularization surgery to treat TIA’s or IS’s, are presented in Table 1. 

**Table 1:** Summary of the obtained data.

Gender		
Men	68%	
Women	32%	
Age	72 (DP = 10)	
Risk factors		
Systemic arterial hypertension	53.3%	
Coronary artery disease	46.6%	
Hyperlipidemia	30.0%	
Active smoking	26.6%	
Diabetes mellitus	23.3%	
Focal symptoms	Initial	Persistent
Amaurosis fugax	7	-
Aphasia	5	2
Dysphasia and or dysarthria	7	4
Upper limb monoparesis	13	5
Hemiparesis	10	3
Hemiplegia	3	3
Non-Focal Symptoms		
Lipothymia	1	
Mental confusion	1	
Transient global anemia	1	
Syncope	8	
Associated with focal symptoms	9	
Carotid disease		
Atheromatous plaques	98%	
Dysplastic plaques	2%	
Constitution of the plaques		
Fibrolipidic and ulcerated plaques	29,2%	
Smooth fibrolipidic	25%	
Smooth calcified plaques	25%	
Calcified and ulcerated plaques	12.5%	
Fibrolipidic, calcified and ulcerated	4.2%	
Fibrodysplastic	2.2%	
Plaques with associated ulcerations	45.8%	
Degree of stenosis quantified		
60 to 70%	4.8%	
70 to 90%	35.7%	
More than 90%	59.4%	
Interval		
Less than 24h - 17 patients	34%	
From 24h to 48h - 02 patients	4%	
From 3 to 5 days - 15 patients	30%	
From 6 to 10 days - 12 patients	24%	
From 11 to 14 days - 04 patients	8%	
Carotid revascularization		
Direct surgery	90%	
Angioplasty plus stenting	10%	

This study included a group of 50 patients underwent revascularization surgery to treat TIA’s and IS’s within a period of 71 months (from August 2014 to June 2020). The absence of mortality and/ or worsening of the neurological condition motivated the continuity this observation-exploratory study. 

This study assessed the data of 34 men and 16 women, aged from 40 to 92 years (mean 72, SD 10). Demographics, risk factors, focal and non-focal symptoms, pathology of the carotid disease, constitution of the carotid plaques, degree of carotid stenosis quantified, interval between the index event and effective revascularization ant the carotid revascularization technique data are presented in Table 1. Risk factors were found in all patients, except in one. Sixteen patients (32%) were already taking antiplatelet agents to treat atheromatosis in other territories that not the carotid region (the majority of those due to coronary disease). Transitory ischemic attack (TIA) was the initial symptom in 28 out of the 50 patients. In 12 of those patients the TIA had been reported as recurrent. 

Many patients had simultaneously more than one neurological manifestation which explains the greater number of symptoms than the study cohort. The left carotid was symptomatic in 28 cases while the right carotid in 22 cases. Only one patient that underwent surgical procedure had been submitted to systemic thrombolysis with alteplase, 72h prior to the direct surgery.

According to the modified Rankin scale, the patients were classified as R0: 35.4%; R1: 45.8%; R2: 18.8% and R3: zero.

Except for three patients that presented with amaurosis fugax, all patients were analyzed with CT at the entrance and 25 with DWI. Although CT of the head is considered an excellent method to exclude intracranial hemorrhages, it was not able to show acute ischemic changes in 98% of the cases. On the other hand, contrasted MRI, especially contrasted DWI, showed in changes in the blood flow in 17 out of the 25 cases (68%). The detected ischemic areas varied from a few mm to a maximum of 15mm in diameter. Patients with larger ischemic areas were excluded from this study. The number of ischemic foci was not valued, only their dimensions. Many patients had several foci. Noteworthy, one patient exhibited 27 areas of altered flow detectable by DWI. 

Carotid disease was assessed through physical examination and color Doppler echography in all cases. Atheromatous plaques (98% of cases) or dysplastic lesion (2%) had their degree of stenosis quantified in 42 detailed studies. The results indicated: obstruction from 60 to 70% (4.8%); from 70 to 90% (35.7%); more than 90% (59.4%). Among these, 13 were considered critical (30.9%). Regarding their constitution, information was reported in 24 cases: the fibrolipidic and ulcerated plaques were found in 29.2% patients, smooth fibrolipidic in 25.0%, smooth calcified plaques in 25%, calcified and ulcerated plaques in 12.5%. The plaque was mixed, fibrolipidic, calcified and ulcerated in 4.2% and in another 4.2% the lesion was fibrodysplastic. Ulcerations were found in 45.8%. Most patients were also submitted to studies with computed angiotomography or MR angiography, which were out of the scope of this work.

An early surgical approach was adopted in all 50 patients. For instance, less than 24h after the occurrence of the ischemic event (34%); from 24 to 48h (4%); from 3 to 5 days (30%); from 6 to 10 days (24%); from 11 to 14 days (8%). Carotid revascularization was performed by direct surgery in 90% of the cases and by angioplasty plus stenting in 10% of the cases. In cases of direct surgery, anesthesia was selected according to the surgeon’s preference. Therefore, locoregional anesthesia with conscious sedation was applied in 54% of the patients and general anesthesia 46% of the patients. Hemodynamic monitoring was carried out in all patients. The reconstruction technique varied according to the case: the cases of atherothrombosis were treated either by endarterectomy (CE) or by angioplasty with stenting. Surgical technique in the 40 patients submitted to direct surgery were eversion technique in 22 cases (55%); longitudinal endarterectomy followed by closure with modified bovine pericardium patch in 16 patients (40%) and dacron patch in one case (2,5%). Segmental resection and end-to-end anastomosis were used in the single patient with fibrodysplasia (2,5%).

**Figure 1:**
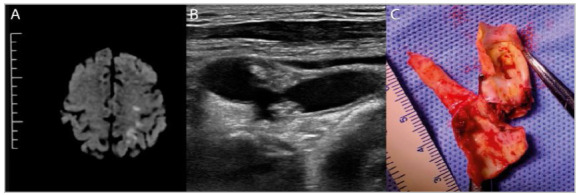
Three images of the same patient with recent ischemic event - A) Diffusion-weighted magnetic resonance study: multiple ischemic areas in the left hemisphere, due to carotid emboli; B) Duplex-can study of the bulb and ICA atherosclerotic vulnerable plaque, with severe stenosis; plaque heterogeneity, disruption of the fibrous capsule and multiple ulcerations are evident; C) Open plaque removed by endarterectomy: the findings of the duplex-study are confirmed - necrohemorrhagic ulcerated plaque, with severe stenosis of the ICA.s.

All patients who underwent surgery under general anesthesia received cerebral perfusion during clamping, using a temporary internal shunt (Pruitt-Inahara™, LeMaitre Medical, Burlington, MA, USA or Javid™, Bard, Tempe, AZ, USA). Two of the 21 patients (9,5%) treated under cervical block needed a temporary indwelling shunt, as they demonstrated cognitive or motor changes during clamping. The Figure 1 summarizes the findings of a MRI - DWI with several ischemic foci of embolic origin (A), the echo Doppler B Mode imaging of the emboligenic carotid plaque and the surgical specimen of the plaque (C). 

In all cases of angioplasty with stenting, the procedure was performed under local anesthesia and conscious sedation, through a femoral approach, using the Seldinger technique. Following a selective catheterization of the targeted carotid artery together with extra and intracranial angiographic studies, embolic protection systems (EPD) were used. In four cases, the Mo.Ma™ device (Medtronic, Santa Rosa, CA, USA) was used to provide flow interruption and in one case, a EZ™ distal protection filter (Boston Scientific, Natick, MA, USA) was employed. In all cases, after installing the EPD, an initial angioplasty was performed (pre dilatation). Thereafter, Casper stents (Terumo, Tokyo, Japan) were implanted when the Mo.Ma device was used and two sequential Wallstents™ (Boston Scientific, Natick, MA, USA), used when the EZ™ filter was employed (for actinic cervical lesion). Post-dilations were not performed.

All treated patients had their cognition and motor skills tested on the operating table. Neither new central neurological deficits nor worsening of existing ones were detected. A cervical hematoma developed in one patient during the postoperative period requiring reintervention. One patient treated with carotid angioplasty and stenting presented a small ipsilateral frontoparietal hemorrhage, unrelated to former ischemic areas. However, an adequate response to conservative treatment was found. At hospital discharge, no additional neurologic deficits were observed. All 50 patients were submitted to a 30-day duplex-scan control study of the operated carotid and a follow-up consultation. All arteries treated were patent and no new neurological symptoms developed in this time interval. 

## DISCUSSION

Stroke is currently one of the leading causes of death worldwide and circa 20 to 30% have an extracranial carotid disease as its major cause^1^. Currently, circa 750,000 strokes occur in the USA yearly. Approximately 15% are immediately fatal, 15-20% are severely disabling and other 15-20% of the cases who were recovering from a former insult, suffer a subsequent incapacitating attack^15^. Figures in Brazil are similar, with 250,000 strokes reported yearly^6^ ^,^ ^16^. Historical studies confirm that symptomatic patients with carotid stenosis in the 50 to 90% range have a high incidence of a new event within seven days of the index stroke^17^.

Tsantilas et al., just to quote one of the most extensive papers on this subject, published a systematic revision with the objective to correlate the early risk of IS after a TIA, amaurosis fugax or non-incapacitating stroke, in patients with 50-99% symptomatic carotid stenosis. Ten publications from 1950 to 2015 were selected. From this analysis, the recurrence is high, corresponding to 6,4% (1.5-23) for a new event in 2-3 days, 19.5% (12.7-28.7) in 7 days and 26.1% (20.6-32.5) for a period of up to 14 days^18^. 

In regard of these publications, we can affirm that after a cerebrovascular index event the patients are at high risk of a definitive stroke, confirming the need of an effective therapeutic approach as soon as possible^18^ ^,^ ^19^.

In the sixties, a surgical approach was indicated for acute stroke with therapeutic objectives in many cases: the results were catastrophic in most instances^20^. In the 80s and 90s a preventive approach was instituted, with better results, but excluding all acute cases^15^ ^,^ ^20^. Since then, many determinants of the success related to carotid revascularization were used to treat ischemic stroke, mainly based in a time interval. Weeks and even months has been proposed as the ideal time interval to perform the surgical procedure after an ischemic cerebral event^20^. 

More recent publications, however, confirm that acting more quickly is important to reduce the damage to the nervous tissue^21^ ^-^ ^28^. However, the variables that determine a safe approach have not yet been definitively established.

The importance of the extension of the ischemic lesion has gained interest recently. Studies dedicated to this theme are few. In 2004, Paty et al. reviewed charts of patients treated from 1980 to 2001, and when CT’s and MR’s studies were available, correlated the size and location of the infarct size with NASCET criteria of indications of carotid surgery^21^. A direct correlation between the preoperative infarct size and the risk of permanent neurological deficit was confirmed: the risk of a permanent neurological deficit raised in a proportion of 1,73% for each 1 cm growth in infarct size. They concluded that the risk of a permanent post operative deficit could be based in the preoperative brain infarct size^21^.

Battocchio et al. identified that emergency treatment (less than 48h) could be performed in patients with minor cerebral damage. Their cut-off was an infarct size of 2,5cm. They treated 16 patients in this group, with good results in 87%^22^. Ferrero et al. evaluated 176 patients with TIA,s and IS’s submitted to CE in up to 48h after the index event. Ischemic sizes of 3cm or less in CT or MR studies and mRs <4 were the cut-offs. The incidence of IS after early CE was 3,4% (six cases); CT and MR confirmed extended or new infarcts in all these cases. In this study, the cumulative risk of TIS, IS and death was 3,9%^23^. Capoccia et al. studied with CT or MR 62 patients with TIA’s or IS’s. Brain infarct size was 33% of the brain supplied by the middle cerebral artery (MCA), with a median of 1,2cm of infarct size. Carotid endarterectomy was performed in less than 6h in 22 patients and in more than this time span in 40. In this series, no new neurologic deficits were identified; in the patients that did not improved after CE, no new lesions were found and the worsening was attributed to brain edema^24^. In later publications, Capoccia et al., Gajin et al. and Dorigo et al. reported that selected patients with infarcts compromising less than one third of MCA territory could be revascularized with death rates ranging from 2-8% for IS’s and of 0-2% for TIA’s in crescendo^25^ ^-^ ^27^.

In contrast with the 2018 European Society of Vascular Surgery Guidelines, that recommended that patients who suffered from IS’s should be submitted to CE later, due to the risk of hemorrhagic transformation of the ischemic areas, the latest Guidelines of the same Society (2023), states that CE confers maximum benefit if performed within 14 days of symptom onset, in patients with mRs up to 3^19^ ^,^ ^28^ ^,^ ^29^. Patients presenting with mRS >4 and infarcts of more than one third of the MCA territory and conscience disturbances, should not be submitted to carotid revascularization before neurological stabilization. These Guidelines are unclear regarding smaller infarct sizes but recommend intervention within 24 hours in strokes-in-evolution and in crescendo TIA’s^28^.

Based on the data obtained from the above quoted articles (References 21 to 28), we observed an increasing post operative risk of stroke and death in ischemic areas larger than 17mm in its largest measurement, as determined by DWI. So, in this study, we limited the indication for revascularization in patients with ischemic changes up to 15mm.

To carotid revascularization be safely performed, patients must have a strict hemodynamic control in the pre-, per- and postoperative period and the variables stroke or TIA as the index event, the severity of the clinical symptoms at the moment of admission, assessed through the National Institute of Health Stroke Scale (NIHSS) or mRs. The extent of the parenchymal lesion evaluated with image exams must be analyzed on a case-by-case basis^10^ ^,^ ^30^. Such strategy allows surgeons to perform the revascularization with the most feasible techniques and that better fits to the needs of each patient.

For this assessment, we must use all the available diagnostic tools. Moreover, the presence of a specialized multidisciplinary team is mandatory. Specialized neurologists are crucial professionals for clinical evaluation and classification of NIHSS or Rankin scores as well as during the screening of patients who may undergo surgical treatment. Radiologists also play a crucial role in this process, since imaging exams identify the criteria used in the therapeutic approaches. CT evaluates the presence or absence of hemorrhagic injury^29^ ^,^ ^30^). On the other hand, Doppler echography is responsible for quantifying the degree of the arterial stenosis and the constitution of the carotid plaque while DWI acutely identifies the ischemic areas within the cerebral parenchyma^15^ ^,^ ^19^ ^,^ ^28^ ^,^ ^31^. Furthermore, angiotomography assesses the anatomy of the circulation from the aortic arch to the brain. A vascular surgery team must be accessible and trained in order to provide an adequate evaluation and immediate intervention of each patient, when indicated^19^ ^,^ ^21^ ^-^ ^28^. 

The examination of the available scientific literature reveals that the two most important factors to establish the ideal moment of carotid revascularization after recent stroke are determined by the patient’s neurological status at the time of admission and the presence or absence of parenchymal lesion its extent and nature. These are evidenced by modern imaging methods, especially CT and MRI^28^ ^,^ ^31^. 

Our results prove that carotid revascularization can be achieved without morbidity and mortality in patients with a modified Rankin score <2, absence of intracranial hemorrhage and presence of ischemic areas not larger than 15mm in diameter. In a future study, revascularization may be evaluated in larger ischemic areas.

## CONCLUSION

The findings of the current study confirm that the size of the ischemic lesion is a more reliable determinant to safely perform the carotid revascularization after an ischemic insult than the time interval from the index event, as broadly accepted. In the 50 patients with TIA’s and IS’s of this study, carotid revascularization was performed in the shortest possible time after the event, based on the extension of the ischemic area. It was both safe and effective in preventing new neurologic deficits, or worsening the ones already installed.
